# Hysterical Spasms Cured with Pulsatilla

**Published:** 1890-08

**Authors:** Paul Lutze

**Affiliations:** Coethen


### HYSTERICAL SPASMS CURED WITH PULSATILLA[30].

DR. PAUL LUTZE, COETHEN.

A young woman complained that for the last nine months she suffered from periodical spasms, during which she is forced to scream out. Accidentally the doctor had the opportunity to witness one or two during his office-hours. It sets in with weak, but gradually increasing respiratory troubles, similar to the puffing of a locomotive, followed by heartrending screams, accompanied by thrashing about; consciousness seemed abolished during these attacks, which is rather a rare symptom in hysteria. They usually appeared toward five P. M.; menses scanty and weak, mostly only every six or eight weeks. In consequence of a former endocarditis there was some slight mitral deficiency. The symptoms indicated Pulsatilla[30], which he prescribed in the manner of his dosage in chronic affections: to take of a watery solution a teaspoonful mornings and evenings for three days, then a free interval of eleven days, so that the four powders (second and fourth placebos) sufficed for four weeks. During the first two weeks the fits increased (medical aggravation?) but the last two weeks no fit. Courses appeared regularly after four weeks. Then the fits returned every other day, which might be considered a critical aggravation, sometimes observed before a final one is obtained. Pulsatilla[30] as before. Three months afterward, during which she took placebos, she had no fits any more, and they never returned, and as he treated her since for a dry herpes on the chin, the woman can be considered cured.—*All. Hom. Zeit.*

---

The case would be hardly worth while publishing if it were not for the manner in which Lutze prescribed the drug. Morning and evening a minute dose of the 30th, and then nearly two weeks tincture of time and patience are the order. Yet we see even that this close prescriber erred in repeating his remedy, when everything hinted to a speedy cure. I once heard my valued friend, Professor Carleton, remark at a meeting of the New York County Medical Society that waiting for the action of a remedy is the greatest trial for a conscientious prescriber, and that we often fail by being in too much of a hurry. How few practitioners know the duration of a drug in susceptible persons, and when life-force is once set in motion in order to restore the equilibrium, any repetition or change can only act in a disturbing manner. Instead of bio-chemistry, let us rather study the biological laws, as lately shown by Arndt and Schulze.

